# EXPLORING FUNCTION OVER TIME IN OLDER ADULTS WITH ADVANCED HEART FAILURE

**DOI:** 10.1093/geroni/igz038.1914

**Published:** 2019-11-08

**Authors:** Theresa Floegel, Alexander Schoemann, Catherine Taylor

**Affiliations:** East Carolina Unviersity, Greenville, North Carolina, United States

## Abstract

Heart failure contributes to functional limitation in older adults, which is associated with rehospitalization and poorer health. However little is known about the trajectory of function after hospitalization in this population or its’ association with readmission. Objective measurement over time to assess physical function trajectory in older adults with heart failure is needed. Purpose: To explore trajectory of function in older adults with heart failure who have history of hospitalization. Methods: Exploratory longitudinal design. Bi-weekly visits were conducted over 4 months with home-dwelling, ambulatory adults with heart failure (60+ years, NYHA classification III) who had a recent acute hospitalization. Function was assessed by Short Physical Performance Battery (SPPB) test and hand-grip strength (9 time-points). Descriptive analyses across time and groups comparison were performed. Results: Participants (N=10) were 75.3±4.6 years (2 female, 5 African American), with EF(%) 39±10 and 8.2±2.9 comorbidities. Half (n=5) were re-hospitalized during the study period. Those re-hospitalized scored an average 1.3 points lower on SPPB and had 2.03 kg less hand strength over time than those not re-hospitalized. An SPPB score <6 was observed across time in 51% of those re-hospitalized, compared to 31% of those remaining at home. Intra-individual function varied but did not decline over this time period. Conclusions: In a sample of older adults with heart failure, physical function ranged from some level of functional disability to functional dependence, with higher levels of disability observed in those re-hospitalized. A longer study period and larger sample may be needed to adequately assess physical function trajectory.

